# Synaptic Plasticity Abnormalities in Fetal Alcohol Spectrum Disorders

**DOI:** 10.3390/cells12030442

**Published:** 2023-01-29

**Authors:** Balapal S. Basavarajappa, Shivakumar Subbanna

**Affiliations:** 1 Center for Dementia Research, Nathan Kline Institute for Psychiatric Research, Orangeburg, NY 10962, USA; 2 Molecular Imaging and Neuropathology Area, New York State Psychiatric Institute, New York, NY 10032, USA; 3 Department of Psychiatry, Columbia University Irving Medical Center, New York, NY 10032, USA; 4 Department of Psychiatry, New York University Langone Medical Center, New York, NY 10016, USA

**Keywords:** alcohol, pregnancy, receptors, neurotransmitter, gene expression, protein expression, synaptic structure, learning and memory, cognitive behavior, intellectual disabilities

## Abstract

The brain’s ability to strengthen or weaken synaptic connections is often termed synaptic plasticity. It has been shown to function in brain remodeling following different types of brain damage (e.g., drugs of abuse, alcohol use disorders, neurodegenerative diseases, and inflammatory conditions). Although synaptic plasticity mechanisms have been extensively studied, how neural plasticity can influence neurobehavioral abnormalities in alcohol use disorders (AUDs) is far from being completely understood. Alcohol use during pregnancy and its harmful effects on the developing offspring are major public health, social, and economic challenges. The significant attribute of prenatal alcohol exposure on offspring is damage to the central nervous system (CNS), causing a range of synaptic structural, functional, and behavioral impairments, collectively called fetal alcohol spectrum disorder (FASD). Although the synaptic mechanisms in FASD are limited, emerging evidence suggests that FASD pathogenesis involves altering a set of molecules involved in neurotransmission, myelination, and neuroinflammation. These studies identify several immediate and long-lasting changes using many molecular approaches that are essential for synaptic plasticity and cognitive function. Therefore, they can offer potential synaptic targets for the many neurobehavioral abnormalities observed in FASD. In this review, we discuss the substantial research progress in different aspects of synaptic and molecular changes that can shed light on the mechanism of synaptic dysfunction in FASD. Increasing our understanding of the synaptic changes in FASD will significantly advance our knowledge and could provide a basis for finding novel therapeutic targets and innovative treatment strategies.

## 1. Introduction

Alcohol is the most regularly abused substance in the world. A recent report from the National Survey on Drug Use and Health indicated that approximately 70% of people aged 18 or older reported alcohol use within the past year [1]. Additionally, nearly 25% of this population engaged in binge drinking (consuming five drinks or more in men and more than four drinks in women within approximately 2 h) within the last month [1]. Despite public education endeavors and recommendations to avoid alcohol use while pregnant, alcohol use by pregnant women remains widespread [2,3,4,5,6,7]. The use of alcohol during pregnancy in the USA has also soared over the years [3,7].

The impact of alcohol abuse during pregnancy on the developing fetus has received extensive attention since the first finding indicating the devastating and persistent complications of fetal alcohol exposure [8]. Among the potential negative consequences of fetal alcohol exposure, brain maturation impairments, followed by lifelong physical, emotional and behavioral problems, are prominent. The major outcome of the effects of high-dose alcohol exposure during early development is fetal alcohol syndrome (FAS) [9]. Pre- and postnatal abnormal growth, craniofacial defects and long-lasting behavioral difficulties characterize FAS. FAS is observed in 1–2 of every 1000 newborns [10]. In addition, FAS has been identified as a significant cause of nongenetic intellectual disabilities and behavioral difficulties worldwide [11,12,13,14,15]. Nonetheless, FAS is not the only enduring abnormality stemming from developmental alcohol exposure. The term fetal alcohol spectrum disorders (FASDs) [16] has been used to exemplify the range of persistent structural and behavioral impairments of developmental alcohol effects, with FAS on the extreme side of the spectrum. The prevalence of FASDs is estimated to be as high as 2–5% [17,18]. In addition, the current literature suggests that severe neuropsychological impairments, such as verbal learning/recall abilities, learning, and memory, characterize FASD [13,19,20,21,22,23]. These neuropsychological deficits have also been associated with most intellectual disabilities in the Western world [21,23,24,25] and impose many daily challenges for children with FASD. The severity of the neurobehavioral outcome of FASD differs and depends on the abuse patterns (continuous versus binge drinking), amount [26,27], and developmental timing [28]. Effective treatments for FASD are currently lacking because the mechanisms underlying alcohol-induced brain damage and neurobehavioral impairments are poorly defined. However, changes in the expression of genes and proteins [29,30,31,32,33,34,35,36,37,38,39,40,41,42,43,44,45,46,47,48] related to neuronal survival, neuronal growth and development, neurotransmission, myelination, synapse formation, and dendritic spines have been suggested to play a significant role in alcohol neurotoxicity. Notably, synapses maintain their molecular composition, plasticity, and function through protein homeostasis, and alterations in such homeostasis may lead to persistent neuronal plasticity deficits.

### Molecular Basis of Neuronal Plasticity

Neuronal plasticity is an essential process that regulates neuronal activity by enabling neurons to fine-tune their synaptic strength in response to changes in activity. This ability is essential during development when circuits are fine-tuned by selective pruning and synapse remodeling in response to experience [49]. By altering synaptic strength, the nervous system can remodel itself, creating long-lasting memories that form the biological basis for brain function. Uncovering the mechanisms regulating synaptic plasticity will help to illuminate how neuronal plasticity disruptions influence the disorder’s pathophysiology, identify new therapeutic targets, and reveal potential impacts of pharmacologically targeting neuronal plasticity.

A central component of plasticity includes the temporal coexistence of activity. Supposedly, spike-timing dependent plasticity is a Hebbian learning rule in which the changes in synaptic strength rely on the relative timing of action potentials [50]. In monosynaptic pairs of neurons, if an output spike from postsynaptic neurons follows immediately after an input spike from the presynaptic neuron, that input becomes stronger, causing long-term potentiation (LTP). If the input spike arises immediately after an output spike, conversely, that input is formed weaker, causing long-term depression (LTD) [51,52,53]. Although STDP primarily involves molecular changes at the synapse, it depends on various distinct mechanisms that can vary in different brain regions, among different neurons within the same brain region, or among similar types of neurons. This critical window of timing dependency covers tens of milliseconds. It has profound consequences on the brain function, producing an activity-dependent bidirectional modification of synaptic strength and eventually establishing the physiological basis for learning and memory. The mechanism underlying STDP involves two different glutamate receptors that are frequently coexpressed, the α-amino-3-hydroxy-5-methyl-4-isoxazolepropionic acid receptor (AMPAR) and the N-methyl-D-aspartate receptor (NMDAR) [54]. The preexisting membrane depolarization of the NMDAR is sequentially mediated through the coactivation of the AMPAR [55] to establish mutual cellular mechanisms that enable long-term synaptic changes.

## 2. Influence of Developmental Alcohol on Neuronal Plasticity

There is growing evidence that neuronal plasticity is persistently impaired in animal models of FASD. Several laboratories have reported neuronal plasticity involving glutamatergic, GABAergic, and their modulator defects in different brain regions using various animal models of FASD and analyses. Here, we discuss the current understanding of these events, emphasizing pre- and postnatal alcohol studies that have contributed to the FASD field under each section.

### 2.1. Developmental Alcohol Influence on the Glutamatergic Neurotransmitter System

**Figure 1:**
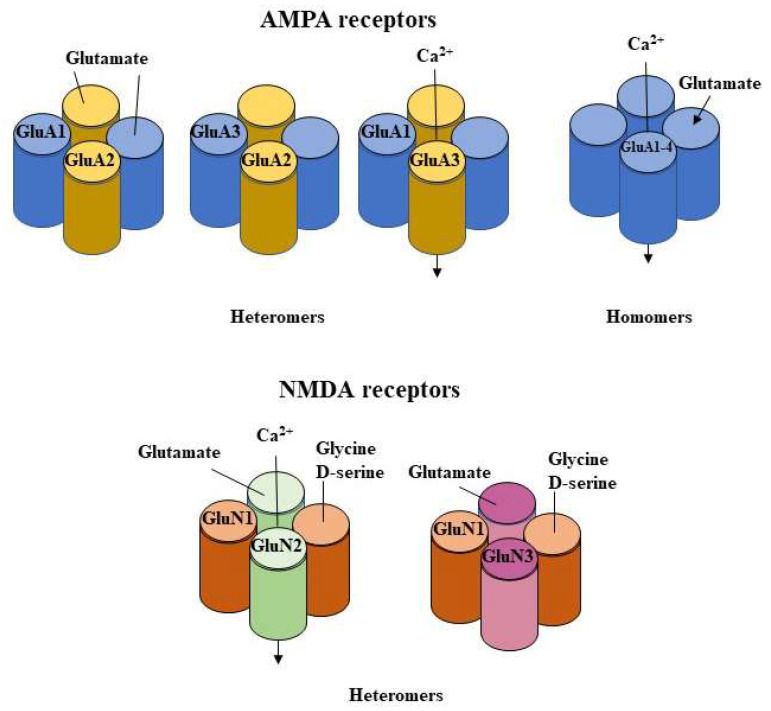
The diagram illustrates the structural diversity of AMPA and NMDA receptor subunits. Multiple subunits have been characterized in these receptor classes that bind either glutamate or glycine/D-serine. Subunits of AMPA receptors can form functional homomers and heteromers, but GluA2-containing heteromers are the most common in the brain [86]. AMPA receptors are classified as GluA2-containing (Ca^2+^-impermeable and insensitive to polyamines) and GluA2-lacking (Ca^2+^-permeable and blocked by polyamines). NMDA receptors exist as heteromeric receptors with two GluN1 subunits and two GluN2 subunits (GluN1/2) or two GluN3 subunits (GluN1/3). NMDA receptor activation requires the simultaneous binding of glycine (or D-serine) and glutamate to relieve the voltage-dependent Mg^2+^ block and allow the flow of inward Ca^2+^ [86].

Glutamate facilitates most excitatory neurotransmission in the mammalian brain by binding to metabotropic glutamate receptors (mGluRs). mGluRs are G protein-coupled receptors and ionotropic glutamate receptors (iGluRs). iGluRs are cation-permeable ligand-gated ion channels. mGluR and iGluR activation results in distinct cellular responses on remarkably different time scales. iGluRs are classified into different functional classes, that is, AMPA receptors, kainate receptors, NMDARs, and GluD receptors (delta or *δ* receptors) [56,57] (Figure 1). In certain conditions, iGluR subtypes that classically mediate current responses (i.e., ionotropic signaling) also have the ability to promote intracellular signaling (i.e., metabotropic signaling) through different mechanisms [58,59].

#### 2.1.1. Influence on AMPA Function

Given the multifactorial functions of iGluRs in normal brain function, it is unsurprising that their dysregulation is implicated in several pathophysiological conditions [60,61,62,63]. Indeed, in heterologous expression systems and some neuronal preparations, alcohol has been shown to inhibit AMPAR function when receptor function was studied using agonist application to isolated cells or oocytes [64,65]. Inhibition of AMPAR function was evident in acutely isolated hippocampal cells and cultured CNS neurons from postnatal Day 10–20 mice [66]. These findings suggest that alcohol (10–500 mM) inhibition of AMPAR function was due to the stabilization of receptor desensitization [66]. In another study [67], alcohol (50 mM) significantly depressed AMPA-evoked currents in hippocampal slices derived from neonates (postnatal day (PD) 3–6 but not in those from juveniles (PD21–PD26). Additionally, alcohol (50 mM) significantly reduced AMPA-mediated excitatory postsynaptic currents (EPSCs) in neurons from neonatal but not from juvenile rats. Additionally, alcohol (50 mM) significantly increased the paired-pulse plasticity of the AMPA-mediated current ratio in slices from PD5 rats. Furthermore, acute alcohol (50 mM) also decreased miniature excitatory postsynaptic current (mEPSC) frequency in neurons from neonatal rats. These studies suggest that alcohol reduced the probability of glutamate release at CA3 pyramidal neurons in neonatal rats by inhibiting N-type voltage-gated Ca^2+^ channels (VGCCs) [67]. In another study, alcohol (40 and 80 mM) inhibited AMPAR-mediated field excitatory postsynaptic potentials (fEPSPs) in acute coronal brain slices prepared from PD7–9 rats. Similarly, alcohol (80 mM) also reduced AMPAR-mediated fEPSPs in the presence of an inhibitor of Ca^2+^-permeable AMPARs. In the same study, alcohol (80 mM) inhibited the LTP of AMPAR-mediated fEPSPs [68].

Because hippocampal AMPAR-mediated synaptic transmission occurs during the first two postnatal weeks in rodents and plays a role in network formation and synapse development [69,70], the findings discussed above highlight the critical role of AMPARs in the action of alcohol during early postnatal development. In another study, acute alcohol (30 and 60 mM) depressed mEPSC frequencies in cultured hippocampal neurons derived from PD1 mice [71], indicating that alcohol inhibits glutamate release. These alcohol doses also enhanced anandamide (N-arachidonoylethanolamide, AEA) and 2-arachidonylglycerol (2-AG), two well-known endocannabinoids (eCBs) shown to depress excitatory neurons by binding to cannabinoid receptor type 1 (CB_1_), in cultured neurons [71]. Furthermore, the CB_1_ receptor antagonist (SR141716A) reversed acute alcohol-induced depression of mEPSC frequency. Additionally, drugs that enhance the in vivo function of eCBs blocked alcohol effects on mEPSC frequency. These findings indicate that acute alcohol-enhanced eCBs are responsible for alcohol-reduced glutamate release in these cultured cells from PD1 mice. Interestingly, it has been well established that CB_1_ receptor activation is associated with inhibition of N-type and P/Q-type Ca^2+^ channels [72] consisting of alcohol inhibition of N-type voltage-gated Ca^2+^ channels (VGCCs) [67]_,_ which may occur via CB_1_ receptor activation. There is also evidence indicating that alcohol inhibits glutamate release in other hippocampal regions. It has been shown that alcohol inhibits KCl-induced vesicular FMI-43 [N-(-3-trethylammoniumpropyl)-4-(-(dibutylamino) styryl) pyridinium dibromide] destaining in the CA1 stratum radiatum of PD21–28 rats, and that the effect was inhibited by blockers of N-type and P/Q-type Ca^2+^ channels [73]. Collectively, these findings corroborate the notion that alcohol inhibits glutamate release in pyramidal neurons by activating an eCB/CB1 receptor system that is negatively coupled to N-type and P/Q-type Ca^2+^ channels [71]. In line with these observations, recent studies have implicated CB_1_ function in developmental alcohol-induced defects. For example, CB_1_ receptor antagonist (SR141716A, SR) administration before postnatal alcohol exposure on postnatal Day 7 (PD7) prevented LTP defects in the hippocampus (HP) and cognitive impairments in adult mice [43,44,45,74,75]. Similarly, PD7 alcohol exposure failed to cause LTP and cognitive impairments in global CB_1_ null adult mice [43,44,45,74,75]. Both SR preadministration and use of global CB_1_ null mice prevented PD7 alcohol-induced neurodegeneration in neonatal mice [43,44,45,74,75]. These findings suggest that PD7 alcohol-activated CB_1_ limited NMDA receptor function, causing neurodegeneration and LTP/cognitive defects by inhibiting glutamate release [45].

#### 2.1.2. Influence on AMPA Subunits Changes

Different developmental alcohol studies also examined AMPAR subunit changes and found different outcomes depending on the model, offspring age, and the region measured (Table 1). For example, alcohol exposure of neonatal rats 3 h a day from PD4–9 significantly reduced cortical GluR1 levels in western blot analysis [76]. Alcohol exposure from GD2–67 in pregnant Dunkin–Hartley strain guinea pigs enhanced GluR2/3 density (binding studies) in the cerebral cortex [77]. In another study, alcohol exposure throughout pregnancy in rats caused cognitive deficits in offspring which was correlated with reduced AMPA-mediated mEPSCs in the HP [78]. Furthermore, alcohol exposure from GD8–20 significantly enhanced Ca^2+^-permeable AMPA receptor (CP-AMPAR, GluR3) expression and enhanced the depression of AMPAR-EPSCs, caused synaptic strength and facilitated anti-Hebbian LTP in VTA-DA neurons of alcohol-exposed PD14–84 animals [79]. In addition, enhanced GluR3-positive particles in cytosol/TH-positive dendrites were observed in electron microscopy analysis [79]. Alcohol exposure restricted to between GD10–18 and PD4–14 increased AMPA receptor function in adult (PD74) medial prefrontal cortex (mPFC) layer VI pyramidal neurons [80]. In the same study, the mice exhibited attention deficits. Using the same alcohol administration regimen, alcohol enhanced AMPA receptor subunits (GluR1, GluR2, and GluR3) in the dentate gyrus (DG) region of PD77 rats [81]. In another study, voluntary alcohol exposure from GD1–PD21 in mice decreased the GluR1/GluR2 ratio in adolescent mouse offspring [82].

Furthermore, Ca^2+^-permeable receptors have been shown to help preserve the availability of Ca^2+^ ions necessary for LTP maintenance [83]. These findings suggest that an increase in Ca^2+^-impermeable receptors at the synapse (GluR2-containing) inhibits the probability of action potential generation and therefore affects NMDA-dependent LTP generation, unlike Ca^2+^-permeable (GluR2-lacking) receptors. Although these findings collectively suggest that alcohol exposure during different stages of pregnancy significantly affects AMPA receptor subunits and their function, how these changes contribute to synaptic plasticity and cognitive deficits in offspring warrants further study.

#### 2.1.3. Influence on NMDA Receptors Subunits

NMDARs play a fundamental role in the neuronal plasticity and neurotransmission that regulate the learning and memory process, which is essential to CNS development and function and is also involved in neurotoxicity. NMDARs are Ca^2+^-permeable tetrameric protein complexes (Figure 1) composed of two obligatory GluN1 subunits and two GluN2 (A–D) or GluN3 (A, B) subunits [63]. Each NMDA receptor activation requires membrane depolarization as well as the binding of both glycine (to GluN1) and glutamate (to GluN2) for the opening of the channel pores [63]. In addition, the GluN2 subunits play a critical role in regulating many of the electrophysiological properties of the receptor. While it is generally agreed that alcohol acutely inhibits NMDA receptors, several studies have indicated that sensitivity to alcohol depends on developmental age and brain region [84,85].

Studies in oocytes have suggested that NMDARs containing GluN2A or GluN2B subunits are more sensitive to alcohol than those containing GluN2C/D [87,88]. Alcohol acts as a partial NMDAR antagonist that inhibits NMDAR-mediated EPSCs over a range of concentrations (1–100 mM). Nevertheless, the inhibition remains partial even at higher concentrations (100–500 mM) [67,89,90,91]. Alcohol has been shown to interact with the NMDA receptors GluN1 and GluN2 subunits, regulating both receptor kinetics and alcohol sensitivity, suggesting that the opposite location of these residues influences the ability of alcohol to control receptor activity [92,93,94]. Although these early studies indicated that alcohol directly affects NMDA receptors, the in vivo animal studies that examined NMDAR changes after alcohol exposure during early development have reported contrasting results. These outcomes depended on the animal species used (e.g., rat, mice, or guinea pig), the timing and period of ethanol exposure (during only one human equivalent trimester of gestation, throughout gestation, during pre- and postnatal periods) and ethanol exposure paradigms (gavage, liquid diet, etc.) and the age at which the outcome was evaluated (neonate, adolescent, or adult offspring). Nevertheless, a large body of literature suggests alterations in NMDAR subunit expression and changes in receptor function (Table 1). For example, a liquid diet containing 3.35% alcohol throughout the gestation period reduced NMDA-sensitive [^3^H]-glutamate binding site density in various subregions of the HP at 45 days of offspring age [95]. Similarly, exposure to alcohol from GD2 to GD62 (4 g/kg/day) in pregnant guinea pigs reduced [^3^H]MK-801 binding in the HP of fetuses (GD63) [96]. Prenatal and postnatal alcohol exposure caused a significant reduction in the density of NMDARs. It increased the percentage of high-affinity state (open channel state) relative to low-affinity state (closed channel state) receptors in the cortex and HP [97]. Alcohol exposure throughout pregnancy reduced GluN1, GluN2A, and GluN2B in the barrel field cortex of adult offspring [98]. Additionally, alcohol exposure throughout pregnancy and lactation significantly increased the HP levels of GluN1 and GluN2D mRNAs on PD7 and 14, reduced GluN2C on PD1 and increased [^3^H]MK-801 binding [99]. If exposure was limited to the sole postnatal period (PD4–9) in rats, GluN2A was increased in the cortex at PD21 in rats [100].

Furthermore, alcohol exposure throughout pregnancy reduced the C2-terminal variant postsynaptic (PSD95-immunoprecipitated) GluN2A subunit in PD21 rat offspring [101]. After prenatal ethanol exposure up to PD9, GluN2A was increased at PD10 in the HP without changes in the cortex [102]. In a mouse study, alcohol exposure to GD8 reduced GluN2B mRNA and increased GluN2A mRNA in PD90 offspring [103]. In another study, alcohol exposure from GD2 until GD63 increased GluN1 mRNA levels in both the CA3 and CA1 areas of the HP of near-term guinea pig fetuses (GD63) [104]. Alcohol exposure throughout pregnancy and lactation reduced GluN1 transcripts in PD60 and PD90 offspring HP [105]. Changes in NMDA receptor subunits in response to alcohol exposure during pre- and/or postnatal development in different models are summarized in Table 1.

**Table 1:** Summary of developmental alcohol exposure effects on glutamatergic neurotransmitter system.

Alcohol Model	BAC	Tissue/Region	Effects
**AMPAR function**			
PD4–9; (vapor, 3h/day) (W rats)	330 mg/dL	NC	Reduced GluR1 (PD10) [76].
GD2–67; Oral (4 g/kg) (Pigs)	327 mg/dL, maternal	Cerebral CE	Reduced GluR3 (PD61) [77].
GD3–21; (4 g/kg/day) (SD Rats)	184 mg/dL, maternal	HP	Reduced AMPA-mediated mEPSCs [78].
GD8–20; (3 & 4 g/kg/day) (SD Rats)	281–341 mg/dL, maternal	VTA	Enhanced Depression of AMPAR-EPSCs [78] and increased GluR3 (PD35–60).
GD1–21 (Alc liquid diet) (LE Rats)	60 mg/dL	HP (DG)	Decreased GluR1 (PD90) [106]
GD8–20 (3 &4 g/kg/day) (SD Rats)	281–341 mg/dL, maternal	VTA	Enhanced Ca^2+^-permeable AMPAR& increased GluR3 (PD14–84) [79].
GD10–11 (2 g/kg/day); GD12–18 (4 g/kg/day); PD4–5 (1.5 g/kg/day); PD6–14 (3 g/kg/day) (C57 mice)	255 mg/dL on PD10	mPFC DG	Increased AMPA receptor function (PD74) [80]. Increased GluR1, 2 & 3 (PD74) [81].
GD1–PD21 (10,15 & 20% Alc sol) (C57 mice)	80 mg/dL, maternal	HP	Reduced GluR1/GluR2 ratio (PD30–58) [82]
**NMDAR function**			
GD1–21 (3.35% Alc liquid diet) (SD Rats)	35–40 mg/dL, maternal	Dorsal HP	Reduced NMDA sensitive [^3^H]-glutamate binding (PD45) [95].
GD2–62 (4 g/kg/day) (SD Rats)	269 mg/dL, maternal	HP	Reduced [^3^H]MK-801 binding (GD63) [96].
GD12–18 (5 g/kg/day) (SD Rats)	143 mg/dL, maternal	HP & CE	Reduced [^3^H]MK-801 binding (PD20–22); Increased high-affinity state [97].
PD4–14 (10.2 % Alc diet) (SD rats)	429 mg/dL, PD8	HP & CE	Reduced [^3^H]MK-801 binding (PD20–22); Increased high-affinity state [97].
GD1–21 (6.5% Alc liquid diet (LE Rats)	133 mg/dL, maternal	Barrel field CE	Reduced GluN1, GluN2A & B (PD90) [98].
GD1–PD9 (10% Alc sol (SD Rats)	86–112 mg/dL, maternal	HP	Increased GluN1 & GluN2D mRNA (PD7 & 14) [99]. Reduced GluN2C (PD1) [99]. Increased [^3^H]MK-801 binding [99].
GD3–21 (20–36% Alc liquid diet (SD Rats)	119–138 mg/dL, maternal	HP and FB	Reduced GluN2A and GluN2B (PD14) in FB; Reduced GluN2B (PD7) in HP [107].
PD4–9 (6.2 g/kg/day)	307 mg/dL (PD4–9)	CE	Increased GluN2A (PD21) [100].
GD1–21 (20–36% Alc liquid diet (SD Rats)	120–145 mg/dL, maternal	Cerebral CE	Reduced cell surface c2-terminal variant postsynaptic GluN2A (PD21) [101].
GD (5 g/kg) & PD4–9 (6.2 g/kg) (SD Rats)	95 mg/dL, maternal; 35 mg/dL, pup	HP	Increased GluN2A (PD10) [102].
GD2–67; Oral (4 g/kg) (Pigs)	327 mg/dL, maternal	WB	Reduced GluN2B (PD61) [77].
GD8; 25% alc, i.p. (C57 mice)	ND	WB	Reduced GluN2B mRNA; increased GluN2A mRNA (P90) [103].
GD2–63; Oral (4 g/kg) (Pigs)	283 mg/dL, maternal	HP	Increased GluN1 mRNA (GD63) [104].
GD1–PD14; 10% Alc sol (SD Rats)	86–112 mg/dL, maternal	HP	Reduced GluN1 mRNA (PD60&90) [105].

ND, not determined.

Concerning GluN1 subunit expression, some studies report it to be increased in the DG of the HP [31], whereas others did not find any changes [108]. The latter authors also reported no changes in GluN2A and GluN2B subunit expression in the DG. Others found that GluN2B was decreased at PD7 in rats or adult mice, accompanied by a decrease in PSD95–GluN2B complex association [109], whereas GluN2B increased in some regions of the prefrontal cortex (PFC) [110]. Interestingly, GluN2B expression was reduced in the DG [30]. At the same time, it was increased in CA1 [111] and caused enhanced LTD, revealing the substructure alterations induced by alcohol. Finally, learning deficits in HP-dependent tasks in adult rats after postnatal alcohol exposure were accompanied by dysregulation in HP gene expression through a significant reduction in glutamate-related genes, including those for GluN2B and GluN2D subunits [112]. Developmental alcohol influence on changes in NMDAR subunits may contribute to synapse dysfunction because the inflow of Ca^2+^ through this receptor is highly dependent upon its subunit composition [61]. In particular, the amount of Ca^2+^ inflow across NMDARs is higher in GluN2B-containing receptors than in GluN2A-containing receptors. Additionally, bidirectional synaptic plasticity is dependent on the postsynaptic Ca^2+^ concentration [113]. Alcohol exposure using a liquid diet from GD3 until birth caused no change in the forebrain PSD-95-associated NMDA-receptor complex in PD1 rats [114]. Reduced whole brain GluN2B mRNA was reported in adult offspring exposed to alcohol on GD8 [103]. In another study, alcohol exposure (two bottled choices) throughout pregnancy decreased PSD-95-associated GluN2B levels in adult HP. Although the mechanism is unclear, these findings suggest that synaptic GluN2B-containing NMDA receptor concentrations decreased in gestational alcohol-exposed adult offspring [109]. Reduced GluN2B-containing NMDA receptors can affect extracellular signal-regulated kinase (ERK) phosphorylation [115], which was reduced in the HP of alcohol-exposed adult offspring [116]. As activation of the Erk1/2 signaling pathway through NMDA receptors [117] is required for various forms of neuronal plasticity [118,119], these observations have relevance in developmental alcohol-induced LTP deficits [120,121] and HP-dependent learning and memory impairments [122].

#### 2.1.4. Influence on NMDA Receptor Functions

Disruption of the PSD-95/GluN2B complex has been shown to reduce cAMP response element-binding protein CREB (a known target of ERK) phosphorylation [123]. Alcohol exposure from GD1 to GD21 caused reduced DG LTP in adolescent males but increased DG LTP in adolescent females [124]. A similar alcohol exposure paradigm also reduced DG LTP in adult males but not in female offspring [108]. These findings indicate that gestational alcohol might have induced neurophysiological alterations during cortical development with increased number and function of NMDARs in females and decreased in males. Consistent with this notion, GD1 to GD21 alcohol exposure enhanced glutamine synthetase expression in the DG with modified excitatory neurotransmission in exposed offspring [108]. In contrast, mice exposed to alcohol throughout pregnancy exhibited a larger NMDA-eEPSC amplitude in the orbital frontal cortex region of female adult offspring. At the same time, reduced NMDA-eEPSC current density was observed in male offspring. In the same study, the contribution of GluN2B subunit-containing NMDARs to eEPSCs was not altered by alcohol. In addition, no change in GluN2B expression in the synaptic fraction of alcohol-exposed males and females was reported [125]. Alcohol exposure throughout the gestational period reduced GluN2B subunit levels and impaired NMDAR-dependent LTP in the DG [30]. Similarly, alcohol exposure from GD3 until PD7 impaired NMDAR-dependent LTP in the CA1 and NMDAR-mediated LTP in the DG of offspring (PD21–60). Moreover, alcohol-exposed offspring rats displayed increased NMDAR-mediated transmission in both HP areas [126]. PD7 alcohol exposure in CD1 mice reduced HP GluN2B levels in males but not in female offspring [127]. In another study, alcohol exposure (10%) during the gestation and lactation period increased GluN2B expression in the synaptic compartment and caused greater low-frequency stimulation (LFS; 600 pulses)-induced LTD compared to control adult CA1 HP slices [111]. In the same study, alcohol exposure reduced LTP. These findings indicate that alcohol exposure during pregnancy increased the highly Ca^2+-^permeable subunit (GluN2B), leading to an increased NMDA-dependent LTD and a concomitantly reduced LTP magnitude.

As discussed above, different alcohol exposure paradigms during the gestational or postnatal period altered the expression of both GluN2A and GluN2B subunits and therefore caused changes in the ratio between these two critical subunits of NMDARs. Interestingly, the GluN2A and GluN2B subunits have been shown to control bidirectional synaptic plasticity in a given neuronal circuit [61]. Especially when the GluN2A-to-GluN2B ratio is low, LTP is more likely to follow through the studied synapse than LTD and vice versa. In this regard, prenatal alcohol exposure in mice caused a decrease in GluN2B subunits and increased C2-containing GluN1 and GluN3A subunits at the HP DG synapse [30]. It should be noted that GluN1 and GluN3 subunits are poorly permeable to Ca^2+^, whereas GluN2B is highly permeable to Ca^2+^. Hence, such changes in subunit expression and the ratio will ultimately modify the plasticity in alcohol-exposed synapses. Alcohol exposure from PD4–9 by gavage reduced the GluN2B subunit in the dorsal HP synaptic compartment, thus increasing the GluN2A to GluN2B subunit ratio [128]. Such changes are thought to be critical for trace fear conditioning [128]. Alcohol exposure (two bottled choices) throughout pregnancy increased GluN1 levels in the synaptosomal membrane fraction without altering GluN2A and GluN2B in the HP DG [109], and PSD-95-associated pools of receptor subunits showed no changes in GluN1 or GluN2A but a decrease in GluN2B [109]. These rearrangements of the NMDA subunits (reduced GluN2B) at the synapse in the DG lead to reduced NMDA-dependent LTP and reduced LTD. Interestingly, in another study, alcohol exposure (10%) during the gestation and lactation period increased the GluN2B subunit in the synaptic compartment in the HP CA1 region [111] due to an increase in the highly Ca^2+-^permeable subunit. These findings suggest that manipulating NMDAR subunit ratios may offer neuroprotection against developmental alcohol-induced neuronal plasticity and may be associated with behavioral abnormalities.

### 2.2. The Developmental Alcohol Effects on the GABAergic Neurotransmitter System

**Figure 2:**
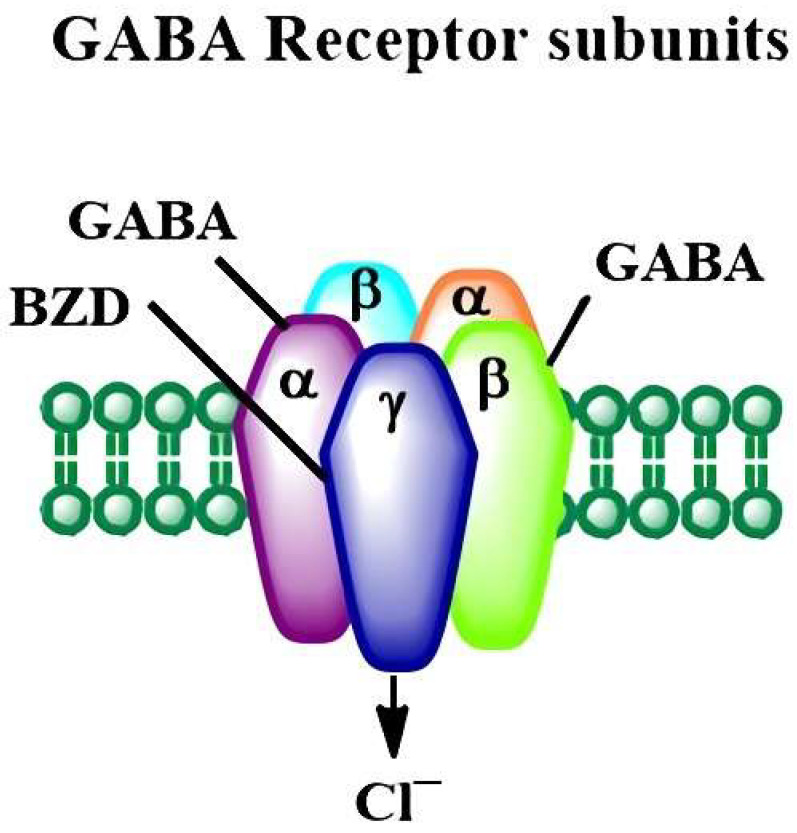
The outline illustrates the structural diversity of GABA_A_ receptor subunits. GABA_A_ ionotropic receptors have been characterized as heteromers consisting of five subunits, most generally two α’s, two β’s, and one γ (α2β2γ). Each subunit consists of many subtypes (α1-6, β1-3, and γ1-3) and exhibits different properties, distributions in the brain, and a wide range of activities in response to pharmacological agents.

Inhibition of neuronal plasticity plays a crucial role in regulating neuronal homeostasis, which is the basis of nervous system function. This inhibition is primarily facilitated by the neurotransmitters GABA and glycine, which activate Cl⁻ permeable ion channels, indicating that the strength of inhibition rests on the Cl⁻ gradient across the synaptic membrane [129]. Therefore, the balance between inhibitory neuronal transmission through GABA and excitatory neuronal transmission through glutamate is indispensable for proper neuronal stability and neurologic function. GABA receptors are classified into GABA_A_ and GABA_B_ [130]. GABA_A_ receptors serve as the major inhibitory neurotransmitter system in the mammalian brain. Each isoform comprises five identical subunits encompassing a central chloride ion-selective channel gated by GABA (Figure 2). GABA_A_ receptors localized to the postsynaptic membrane regulate neuronal inhibition that appears in the millisecond time range, and those localized to the extrasynaptic membrane respond to ambient GABA and are responsible for long-term inhibition [130].

Studies have shown that GABAergic system dysfunction [131,132,133,134,135] likely impacts neurobehavioral abnormalities in offspring exposed to alcohol during development (Table 2). Early studies have explored the influence of gestational alcohol exposure on GABA levels in different brain regions of the offspring. In rats, alcohol exposure (a diet containing 6% ethanol) throughout gestation (4 weeks) increased GABA levels in the cerebral tissues [136]. In rats, alcohol exposure (10%) throughout pregnancy and during the lactation period significantly increased GABA levels in the PFC, olfactory bulb (OFB), anterior colliculus (AC) and amygdala (Amy) tissues from PD21 offspring [137]. The same study found decreased GABA levels in the thalamus, pons, cerebellum, and HP [137]. Alcohol exposure in chick embryos on Days E1–E3 increased glutamate decarboxylase (GAD; a GABAergic neuronal marker) in the E8 embryo’s whole brain [138,139]. In rats, alcohol exposure from GD15 to GD18 increased GABAergic responses in adult frontal and somatosensory cortical neurons [140]. In another study, alcohol exposure using a liquid diet (5% ethanol) throughout gestation caused increased sensitivity of GABA_A_ receptor-stimulated Cl- flux in membrane vesicles prepared from different brain regions, suggesting that such a change in the HP might have contributed to synaptic plasticity defects in adult offspring [131]. In another study, alcohol exposure using a liquid diet from GD0–21 in rats reduced the parvalbumin-expressing GABAergic (PV^+^) interneurons in the medial septum [141] and anterior cingulate cortex [142] of adult rats. In a guinea pig study, alcohol exposure throughout pregnancy reduced the number of GAD^+^ ve cells in the somatosensory cortex of adult guinea pigs [143]. In monkeys, alcohol exposure during the first six or the entire 24 weeks of gestation (one day/week) reduced the number of GABA^+^ ve neurons in the somatosensory and motor cortices of adolescent macaques [144]. In mice, alcohol exposure from GD1–14.5 induced premature GABAergic interneuron tangential migration into the cortical anlage in a 14.5-day-old embryo. In the same study, increased GABA levels and GABA sensitivity of migrating interneurons were observed [145]. In mice, alcohol exposure (binge-type; 5% ethanol) for three days from E13.5 and E16.5 increased the density of median ganglionic eminence-derived interneurons in 16.5-day-old embryos [146]. These alcohol effects persistently increase the number of PV+ interneurons in layer V of the mPFC and potentiate GABA_A_ receptor-mediated synaptic transmission in pyramidal neurons [146]. Other studies have also suggested that early alcohol exposure potentiates the depolarizing effects of GABA_A_ receptors in migrating cells and increases neurogenesis in the medial ganglionic eminence [147,148]. In differentiating human pluripotent stem cell-derived neurons, alcohol exposure (50 mM for 50 days) reduced the transcripts related to GABAergic interneuron specification (i.e., *NPY*, *GSX2*, *SST* and *DLX1-6*) without affecting interneuron numbers [149]. In mice, alcohol exposure throughout the gestation period increased spontaneous inhibitory postsynaptic current (IPSC) amplitude and area in OFC pyramidal neurons [150], impaired behavioral flexibility and altered OFC activity [151]. Taken together, these findings suggest that the equivalent alcohol exposure during the first and second trimesters of human pregnancy impair interneuron proliferation, differentiation, migration, and/or survival, and contribute to the synaptic plasticity, cognitive and social problems seen in adolescents/adults with documented prenatal alcohol exposure.

Alcohol exposure during human pregnancy, equivalent to the third trimester, has also been shown to have detrimental effects on the GABAergic system. In rats, exposure to alcohol vapor between PD2–6 increased the number of calretinin^+^ ve interneurons and reduced calbindin^+^ ve interneurons without affecting PV^+^ ve interneurons in the primary motor and somatosensory cortex in adolescent offspring (P60) [152]. In the same model, these investigators also found reduced PV^+^ ve interneurons in the dendritic tree of the striatum (ST) in adolescent offspring (P60) [160]. In mice, exposure to alcohol vapor during gestation (GD12–19) and the neonatal period (PD2–9) reduced cerebellar interneuron numbers at PD16 [153]. In a mouse study, alcohol vapor exposure from PD2–9 reduced interneuron numbers in the adult mouse HP [154]. In the same study, alcohol vapor exposure at PD7 enhanced the number of interneurons that also exhibited activated caspase-3 staining, suggesting that these interneurons are programmed to undergo apoptotic neurodegeneration [154].

Similarly, in PD7 mice, alcohol exposure reduced the numbers of PV^+^ ve interneurons in the adult mPFC [155], as well as in the HP formation and the pyriform cortex [156,157]. Furthermore, in the same PD7 alcohol exposure model, reduced PV^+^ ve and calretinin^+^ ve interneurons were observed in the adult neocortex [158]. Although these alterations significantly contribute to GABAergic neurotransmission defects, the underlying mechanisms that ultimately contribute to neuronal plasticity and behavioral abnormalities require future investigation. Intriguingly, a recent study using the PD7 alcohol vapor model found neuronal degeneration in PD7 mice through inhibition of neuronal activity via the reduced NMDA receptors functions rather than potentiation of Cl current flow through GABA_A_ receptors [159]. In the same study, findings indicated that acute alcohol exposure has no presynaptic or postsynaptic effect on GABA_A_ receptor-mediated synaptic transmission at RSC neurons [159]. However, electrophysiological recordings in slices from adolescent animals showed enhanced peak amplitudes, asynchronous activity, total charge, and reduced rise times of optically evoked GABA_A_ receptor-mediated inhibitory postsynaptic currents [159]. Thus, any of these changes could contribute to the behavioral abnormalities found in animal models of FASD. Therefore, studies to link these two events should be addressed in future preclinical studies. Based on the above studies, it is clear that developmental alcohol exposure has a variable effect on the GABAergic system depending on the brain region and developmental stage at which analysis was made in addition to the alcohol dose used. Increased GABA interneurons may be attributed to enhanced tangential migration [145]. Tangential migration is controlled mainly by GABA signaling. Therefore, reducing ambient GABA could cause defective GABA interneurons migration, which expresses GABA receptors, a target of alcohol. In addition, alcohol has been shown to potentiate GABA signaling by increasing GABA release and receptor signaling leading to premature migration. In addition to these mechanisms, abundance and differences in GABA interneurons composition in each brain region, chloride efflux, growth factors, and calcium signaling defects can contribute to variable GABA interneuronopathy in a brain region-specific manner, which could account for the synaptic and behavioral abnormalities found in FASD and warrant future investigations in this line of research.

### 2.3. The Developmental Effects of Alcohol on Long-Term Synaptic Plasticity

The current literature indicates that the impact of early alcohol exposure on persistent HP long-term synaptic plasticity (Table 3) is dependent on the alcohol administration paradigm, the developmental period of alcohol exposure, and plasticity induction protocols. Initial observations indicated significantly reduced LTP magnitude in CA1 of young adult male offspring exposed to alcohol during GD1–22 [161]. After this initial study, several investigators examined LTP in the CA1 region using different alcohol exposure paradigms. They reported predominantly reduced LTP in males (See Table 3). However, female offspring exhibited mixed results. For example, alcohol exposure from GD1–22 reduced CA1 LTP in young males and increased it in female offspring [162]. Similar results were observed in another study where alcohol was exposed from GD0–22, and CA1 LTP was performed in male and female young animals [163]. In another study, alcohol exposure from GD1–22 reduced CA1 LTP in male adolescents but not in females [164]. Although the mechanisms responsible for these sex differences are unknown, developmental alcohol-induced changes in the activity of the hypothalamic-pituitary-gonadal axis [165,166,167] may contribute to these dimorphic defects, and future studies to address this are warranted.

A few studies have examined the impact of developmental alcohol on CA1 LTD (Table 3). Reduced LTD in the adolescent CA1 was reported in P7 mice exposed to acute alcohol [169]. However, alcohol exposure throughout pregnancy and lactation (three trimesters) enhanced LTD in CA1 from PD50 offspring [111]. Interestingly, the LTD protocol that failed to induce LTD in controls was able to induce LTD in alcohol-exposed offspring [111]. Lower-dose alcohol exposure limited to only the first two trimesters of gestation failed to affect CA1 LTD in vivo in adolescent male and female alcohol-exposed offspring [180]. In another study, alcohol exposure during GD0–22 reduced LTD in CA1 of male alcohol-exposed offspring. However, it enhanced LTD in females compared to controls [163]. Finally, in a recent study, alcohol exposure was restricted to GD8 and 12 and reduced LTD in adult offspring [172]. Although additional studies are required to explain these sex differences in LTD, these limited findings emphasize that developmental alcohol-induced synaptic plasticity imbalance appears sexually dimorphic.

In the DG, developmental alcohol exposure consistently reduced LTP in male offspring. However, in females, no change or facilitation of LTP was observed (Table 3) in different alcohol exposure paradigms. The DG synaptic plasticity deficits appear to be dependent on the developmental period of alcohol exposure. For example, reduced LTP in male offspring was observed only after the second trimester-equivalent (GD11–21), but not in first- or third-trimester-equivalent exposure [174]. However, in other studies, alcohol exposure from GD1–22 and during the third-trimester equivalent reduced LTP in adolescent offspring [126,178,179]. Few studies have explored LTD in the HP DG region and produced both reduced and enhanced LTD [177,178]. Long-term synaptic plasticity relies on signaling cascades that ultimately control the gene expression that regulates these synaptic activities. Therefore, future studies evaluating the developmental alcohol-induced signaling events and transcription mechanisms mediated by several modulators of glutamatergic and GABAergic neurotransmitter systems can provide a potential relationship between these events.

### 2.4. The Influence of Developmental Alcohol on Modulators of Glutamatergic and GABAergic Neurotransmitter System

Dopamine (DA) has been shown to modulate excitatory and inhibitory neurotransmission through second messenger signaling systems activated upon receptor stimulation and through receptor cross-talk. Based on efferent projections, three main dopaminergic tracts, the mesostriatal, mesocortical, and mesolimbic, have been reported in the mammalian brain [181]. Furthermore, the mesostriatal system predominantly functions in voluntary movement, the mesocortical system is involved in motivation, attention, and behavior, and the mesolimbic system has been shown to regulate emotion and memory [181]. Interestingly, dopaminergic projections make synaptic connections with GABAergic neurons in the nucleus accumbens (NAc), PFC, and ST. Likewise, dopaminergic neurons also make synaptic connections with glutamatergic neurons in the PFC, ST, and NAc [181]. DA hypofunction has been observed in different animal models (Table 4), suggesting that the dopaminergic system is sensitive to developmental alcohol in certain brain regions [182,183,184,185,186,187,188]. In a rat study, alcohol exposure from GD6–20 caused supersensitivity of somatodendritic DA autoreceptors in the ventral tegmental area (VTA), as measured by extracellular recording [189]. In a similar study, alcohol exposure from GD8 until parturition produced a long-lasting reduction in DA receptor function distinct from the somatodendritic impulse-regulating D2 autoreceptors [190]. In another rat study, alcohol exposure during GD8–20 reduced the number of spontaneously active VTA DA neurons without altering the firing rate or firing pattern [191]. Later, it was found that impaired postnatal development contributed to a persistent reduction in the spontaneous electrical activity of VTA DA neurons in adult animals [192]. The reduction in the spontaneous electrical activity of VTA DA neurons in alcohol-exposed adult offspring was due to altered inputs to VTA DA neurons [193]. In rats, alcohol exposure from GD8–20 enhanced GluA3 subunits in VTA DA neuron (2–12-week-old) dendrites and increased excitatory synaptic strength and the induction of Ca^2+^-permeable AMPA receptor-dependent LTP, an anti-Hebbian form of LTP [79]. Similar alcohol exposure in rats (GD7–GD20) enhanced basal synaptic transmission [194] and increased presynaptic glutamate release via enhanced D1 receptor function in the corticostriatal pathway in PD30 offspring [194]. These findings suggest that the imbalance between the function of D1 and D2 receptors, resulting from the upregulation of D1 receptors and downregulation of D2 receptors, promoted LTP instead of LTD in alcohol-exposed offspring [194]. Additionally, DA has been shown to bidirectionally regulate GABA_A_ receptor-dependent synaptic transmission by increasing local interneuron excitability through D1 receptors [195] and decreasing quantal GABA release onto pyramidal neurons through a DA type-3 receptor (D3R) [196]. Accordingly, in the rat model, alcohol vapor exposure during PD2–12 decreased DA levels, attenuated D1 receptor-mediated potentiation of sIPSCs and impaired D3R-mediated suppression of mIPSCs in adolescent offspring (PD40–50) pyramidal neurons [197] without affecting D1 and D3 receptor expression. These observations suggest that alcohol exposure during the first and second trimesters of human pregnancy’s equivalent developmental period influences neuronal plasticity via the DA system in adult offspring.

CB_1_ receptors are expressed at axon terminals of glutamatergic and GABAergic neuron types throughout the brain, and function by modulating many physiological processes, such as synaptic plasticity, cognitive functions, and affective behavior [207,208]. Postsynaptic release of eCBs on demand and binding to presynaptic CB_1_ receptors suppresses glutamatergic or GABAergic transmission, a process termed eCB acting as a retrograde neurotransmitter [209,210]. The influence of alcohol on eCB/CB_1_ receptors and the role of eCBs and CB_1_ receptors in AUDsin adult animals have been extensively studied (for references, see [211]). However, although CB_1_ regulates glutamatergic and GABAergic function [212], few studies have explored the influence of eCB/CB_1_ on neuronal survival, plasticity and cognitive function in response to developmental alcohol exposure (Table 4) [43,45,74,198].

Studies have demonstrated that PD7 alcohol exposure causes apoptosis, increases the abundance of eCBs, such as anandamide (AEA), and increases CB_1_ expression in exposed PD8 mice [45]. Additionally, PD7 alcohol exposure induced apoptosis in PD8 mice, which was absent in mice treated with a CB_1_ antagonist (SR141716A, SR) before PD7 alcohol exposure and in PD8 global CB_1_-KO mice [45]. Consistently, CB_1_ activation during the early developmental period via exposure to cannabinoids at doses similar to those observed in cannabis users causes delays in the maturation of neurotransmitter systems [213]. These delays result in cognitive defects [214] identical to those found in several specific human developmental disorders [215], including FASD [216]. Moreover, the acute administration of *Δ^9^-*tetrahydrocannabinol** (THC) (the main active constituent of marijuana), which activates CB1 receptors, to P7 rats enhanced the proapoptotic effects of alcohol [217]. Intriguingly, THC coadministered with low-dose alcohol in PD7 rats increased CB_1_ expression in a brain-region-specific manner in PD8 rat brains [217]. Consistent with these observations, a CB_1_-KO genotype reduces the susceptibility of PD7 mice to the neurotoxic effects of low-dose [217] or high-dose alcohol [45]. In addition to these immediate effects of alcohol, we and others have demonstrated persistent neurobehavioral defects in adult mice exposed to PD7 alcohol [43,44,45,156,171,218,219,220]. Neither CB_1_ antagonist-treated PD7 mice nor global CB_1_-KO mice exhibit LTP, spatial memory or spatial recognition memory abnormalities [44,45] as adults. Consistent with these exciting studies, another study using a zebrafish model suggests that activation of CB_1_ using the agonist arachidonyl-2-chloroethylamide resulted in a FASD-like phenotype that was reversible using SR [221].

Additionally, cannabinoid administration exacerbated the teratogenic effects of alcohol in embryonic mice and zebrafish models, and CB_1_ receptor antagonists attenuated these effects [222]. Furthermore, gestational alcohol exposure from the first- to the third-trimester equivalents of human pregnancy in mice resulted in tonically active CB_1_ in DLS projection neurons [199]. Pharmacologically increasing eCB tone in these studies mimicked the effects of alcohol on synaptic transmission and cognition [199]. In another study, alcohol exposure during GD8–20 resulted in tonic eCB signaling in ST neurons [198]. In the same alcohol exposure paradigm, moderate and high levels of alcohol exposure persistently reduced LFS-induced eCB-mediated LTD in VTA DA neurons in young animals (4–10 weeks) [198]. Alcohol vapor exposure in mice caused increased excitability in DLS medium spiny neurons (MSNs) followed by increased eCB tone [199]. In contrast to the above studies, alcohol exposure between GD8–20 impaired tonic eCB signaling and caused anxiety-like behavior in adult rats [200]. These initial findings collectively indicate that alcohol alters eCB/CB_1_ function. However, this effect depends on the exposure paradigm (pre- or postnatal exposure) and the brain regions studied. Altered CB_1_ signaling may influence lasting synaptic, cognitive, and sociobehavioral defects. However, the underlying neural basis through which CB_1_ contributes to these persistent neuronal and behavioral impairments is unknown and warrants future investigation.

The serotonin neurotransmitter, also called 5-hydroxytryptamine (5-HT), has been shown to bind to 5-HT-gated ion channels (5-HT_3_ receptors) or G protein-coupled receptors (5-HT_1_, 5-HT_2_, and 5-HT_4–7_ receptors). The majority of 5-HT neurons are located in the raphe nuclei in the brain stem. Some of the 5-HT neurons project to the spinal cord, and others project to the cortex, HP, and hypothalamus (for a review, see [223]). Glutamatergic [224] and GABAergic [225] neurons provide input to 5-HT neurons and modulate their functions in many brain regions. In addition, 5-HT neurons are found as early as E12 in rodents and are thought to influence cell proliferation, differentiation, migration, and synapse formation [226]. Therefore, the function of the 5-HT system in developmental alcohol exposure has been investigated by many (Table 4), as changes in the 5-HT system could have a vast effect on neuronal circuit maturation and behavioral outcomes. In a rat study, alcohol exposure throughout gestation reduced 5-HT, 5-hydroxyindoleacetic acid (5-HIAA), and 5-HT_1_ receptors in the motor and somatosensory cortex of PD19 offspring [184,201]. A similar reduction in 5-HT immunoreactivity was found in the medial forebrain (MFB) of E15 and 18 embryos exposed to alcohol from GD8–15 [202,203,204]. Another model of gestational alcohol showed reduced 5-HT and its synthesis enzyme, tryptophan hydroxylase (TPH), in the dorsal raphe of 5-week-old offspring [205]. Alcohol exposure throughout the gestation and lactation period reduced 5-HT in the amygdala and cingulate cortex (CC) of offspring (PD77–84), as the 5-HT_1_ receptor was reduced in the CC region and increased in the Amy region [206]. Alcohol exposure between GD8–20 potentiated glutamate synapses of dorsal raphe nucleus-5-HT neurons and caused anxiety-like behavior in adult rats [200]. These findings indicate that developmental alcohol significantly impairs the 5-HT system. However, the mechanisms are less clear and future investigations on the underlying detrimental effects of developmental alcohol on the 5-HT system and its influence on synaptic plasticity and behavioral outcomes are warranted.

## 3. Conclusions

**Figure 3:**
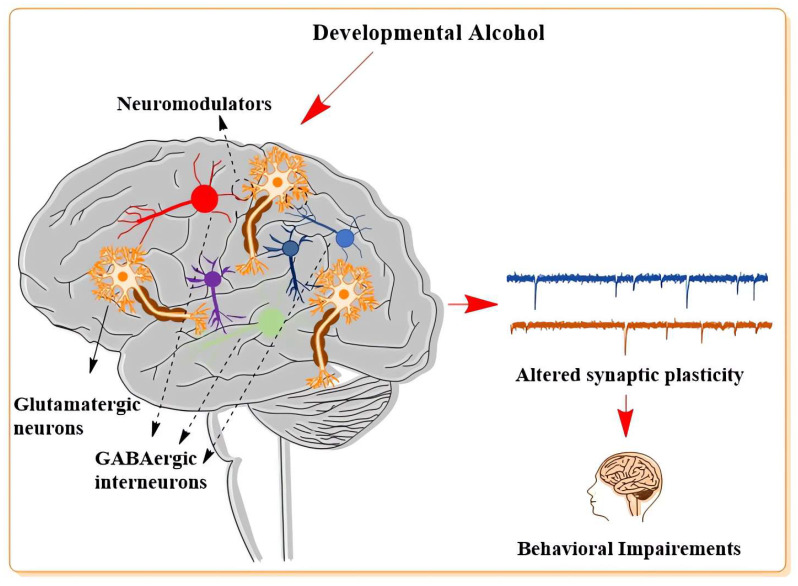
Alterations in glutamatergic and GABAergic neurons and their modulators were found in FASD models. Developmental alcohol exposure causes persistent changes in glutamatergic and GABAergic neuron function, causing synaptic plasticity defects that result in many psychiatric disorders found in FASD, including cognitive and social interaction behavioral impairments.

Investigations have increased significantly over the past decade into the influence of developmental alcohol exposure on neuronal plasticity mediated by glutamatergic and GABAergic systems and modulators of these two critical neurotransmitters. These studies were made possible thanks to technical and conceptual advances in the neuroscience field over the past decade. Studies have clearly revealed that these neurotransmitter systems in the developing brain are highly vulnerable to alcohol exposure compared to the adult brain. This is because components of developing neurotransmitter systems have unique features that make them specifically sensitive to the harmful effects of low- to high-dose alcohol exposure. A great deal of literature (Table 1, Table 2, Table 3 and Table 4) has demonstrated that developmental alcohol exposure causes defects in the formation and refinement of neuronal circuits/assemblies mediated by glutamatergic, GABAergic, and their modulator signaling events (Figure 3), which are likely to contribute, in part, to the long-lasting structural and functional brain impairments that feature FASD. Neuronal plasticity defects are eventually responsible for the behavioral and cognitive outcomes observed in children with FASD and their increased tendency to exhibit comorbid neuropsychiatric and neurological disorders. In future studies, the underlying mechanisms by which developmental alcohol alters glutamatergic and GABAergic systems should continue to be evaluated, and these studies should be expanded to other modulators of neurotransmitter systems (DA, eCB/CB_1,_ and 5-HT) which may provide the therapeutic potential to treat FASD.

## Figures and Tables

**Table 2:** Summary of developmental alcohol exposure effects on GABAergic neurotransmitter system.

Alcohol Model	BAC	Tissue/Region	Effects
GD1–14 (Alc liquid diet) (A Rats)	ND	WB	Increased GABA (GD18&21) [76,136].
GD1–PD14 (10% Alc sol, Oral (WS Rats)	360 mg/dL (PD21)	THA, Pons, CBL, FC, OLB, AC	Increased GABA (FC, OLB, AC&Amy); Reduced GABA (THA, Pons, CBL&HP); (PD21) [137].
E1–E3 (10 mg/50 µL) (Chick embryo)	12 mM	WB	Increased GAD activity (E8) [138,139].
GD15–18 (2.4 g/kg/4 times/day) (SD rats)	ND	Cortical neurons	Increased GABAergic responses (PD90) [140].
GD1–20 (5% Alc liquid diet) (SD Rats)	83 mg/dL, maternal	mFC&HP	Increased GABA_A_ receptor-stimulated Cl-flux [131].
GD0–21 (Alc liquid diet) (LE Rats)	161 mg/dL, maternal	MS&ACC	Reduced GABAergic (PV^+^) IN (PD60) [141,142].
GD2–67 (4 g/kg, oral) (Pigs)	327 mg/dL, maternal	SC	Reduced GAD+ cells (PD220) [143]
GD3–42 &3-168 (1.8 g/kg/day, intragastric) (Pigs)	234 mg/dL, maternal	SC and MC	Reduced GABA^+^ ve neurons [144].
GD1–14.5 (Alc liquid diet) (C57 mice)	25 mg/dL, maternal	Corticle anlage	Premature GABAergic interneuron tangential migration; Increased GABA and GABA sensitivity [145].
GD13.5–16.5 (5% Alc liquid diet) (C57mice)	80 mg/dL, maternal	Embryo and adult mPFC	Increased median ganglionic eminence-derived IN (E16.5) [146]; Increased PV+ IN (mPFC) & potentiated GABA_A_R transmission (PN) [146].
GD13.5–16.5 (5% Alc liquid diet) (Nkx2.1-Cre mice)	80 mg/dL, maternal	PFC	Increased depolarizing action of GABA_A_R in migrating neurons (PD58) [146,147].
GD1–20 (4 g/kg/day) (C57 mice)	80 mg/dL, maternal	OFC	Increased spontaneous IPSCs amplitude and area in PYNs (PD60) [150].
PD2–PD6 (Alc vapor) (WS Rats)	206 mg/dL, maternal	MC&SC	Increased CR^+^ ve INs; Reduced CB^+^ ve INs (PD60) [152]; Reduced PV^+^ ve Ins (ST) [152].
GD12–19 & PD2–9 (Alc vapor) (Venus-VGAT-mouse line)	330 mg/dL, PD7–8	CBL	Reduced INs (PD16) [153].
PD2–9 (Alc vapor) (Venus-VGAT-mouse)	221 mg/dL, PD9	HP	Reduced INs (PD90) [154].
PD7 (Alc vapor) (Venus-VGAT-mouse line)	297 mg/dL, PD7	HP	Reduced INs (PD90) [154].
PD7 (2.5 g Alc × 2times) (C57 Mice))	500 mg/dL, PD7	mPFC, HP&PC	Reduced PV^+^ ve INs (PD90) [155,156,157].
PD7 (2.5 g Alc × 2times) (C57 Mice))	500 mg/dL, PD7	NC	Reduced PV^+^ ve & CA^+^ ve INs (PD90) [158].
PD7 (Alc vapor) (Venus-VGAT-mouse line)	297 mg/dL, PD7	RSC	Enhancement of PV^+^ ve INs-mediated Neurotransmission (PD40–60) [159].

ND, not determined.

**Table 3:** Summary of developmental alcohol exposure effects on hippocampal LTP and LTD in offspring.

Alcohol Model	BAC	HP Slices	Effects
GD1–22 (Alc liquid diet) (Rats)	31 mg/dL, maternal	CA1	Reduced LTP (PD50–70) [161].
GD2–67 (Oral Alc, 4 g/kg/day) (Pigs)	416 mg/dL, maternal	CA1	Reduced LTP (PD40–50) [168].
PD7 (2.5 g/kg × 2/day) (C57 mice)	500 mg/dL, PD7	CA1	Reduced LTP (PD30–32) [169].
PD2–9 (Alc vapor) (SD Rats)	395 mg/dL, PD9	CA1	Reduced LTP (PD7–9) [170]
GD1–22 (Alc liquid diet) (LE Rats)	87 mg/dL, maternal	CA1	Reduced LTP in males; Increased LTP in females (PD30–35) [162].
GD0–22 (Alc 4 g/kg/day) (W Rats)	ND	CA1	Reduced LTP in males and increased in females (PD36) [163].
GD1–22 (Alc liquid diet) (SD Rats)	135 mg/dL, maternal	CA1	Reduced LTP in males and no change in females (PD55–65) [164].
PD7 (2.5 g/kg/twice) (C57 mice)	490 mg/dL, PD7	CA1	Reduced LTP (PD90) [43,45,74,75,171].
GD8&12 (1.75 g/kg/twice) (C57 mice)	300 mg/dL, maternal	CA1	Reduced LTP (PD90) [172].
GD5–PD7 (10% Alc sol) (SD rats)	ND	CA1	Reduced LTP (PD17–30) [173].
PD7 (2.5 g/kg × 2/day) (C57 mice)	500 mg/dL, PD7	CA1	Abolished LTD (PD30–32) [169].
GD1–22&lactation (10% alcohol sol) (SD rats)	ND	CA1	Increased LTD (PD50–52) [111].
GD0–22 (Alc 4 g/kg/day) (WS Rats)	ND	CA1	Reduced LTD in males and increased in females (PD36) [163].
GD8&12 (1.75 g/kg/twice) (C57 mice)	300 mg/dL, maternal	CA1	Reduced LTD in males (PD90) [172].
GD1–22 (Alc liquid diet) (LE Rats)	84 mg/dL, maternal	DG	Reduced LTP (PD105–140) [162].
GD11–21 (Alc liquid diet)	142 mg/dL	DG	Reduced LTP in males(PD50–70) [174].
GD1–22 (Alc liquid diet) (SD Rats)	135 mg/dL, maternal	DG	Reduced LTP in males and no change in females (PD55–70) [175].
GD1–22 (Alc 5%liquid diet) (SD Rats)	146 mg/dL, maternal	DG	Reduced LTP in males and no change in females (PD55–70) [108].
GD1–22 (5% Alc in water) (LE Rats)	84 mg/dL, maternal	DG	Reduced LTP (PD105–140) [176].
GD1–PD14 (10% Alc sol.) (SD Rats)	100 mg/dL, PD7	DG	Reduced LTP (PD45–55) [177].
GD1–22 (Alc liquid diet) (SD Rats)	80–180 mg/dL, maternal	DG	Reduced LTP in males and females (PD21–28) [178].
GD1–PD7 (10% Alc sol) (SD Rats)	62 mg/dL, maternal	CA1 & DG	Reduced LTP (PD21–60) [126].
GD1–22 (Alc liquid diet) (SD Rats)	80–180 mg/dL, maternal	DG	Reduced LTP in males only (PD31–35) [179].
GD1–PD14 (10% Alc sol.)(SD Rats)	100 mg/dL, PD7	DG	Facilitated LTD (PD45–55) [177].
GD1–22 (Alc liquid diet) (SD Rats)	80–180 mg/dL, maternal	DG	Reduced LTD in females only (PD21–28) [178].

**Table 4:** Summary of the influence of developmental alcohol on modulators of glutamatergic and GABAergic neurotransmitter system.

Alcohol Model	BAC	Tissue/Region	Effects
GD6–20 (alc liquid diet) (LE Rats)	ND	VTA	Supersensitive DA autoreceptors (PD90–120) [189].
GD6–20 (alc liquid diet) (LE Rats)	ND	NSDA	Reduced DA receptor functions (P90–120) [190].
GD8–20 (3 g/kg/twice/day) (SD rats)	281–341 mg/dL, maternal	VTA	Reduced the number of spontaneously active DA neurons (P90) [191].
GD8–20 (3 g/kg/twice a day) (SD rats)	281–341 mg/dL, maternal	VTA-DA	Enhanced GluA3 (PD14–84) [79]; Enhanced EPSCs strength (PD14–84) [79].
GD7–20 (6g/kg/day) (SD Rats)	302–331 mg/dL, maternal	DL-ST	Enhanced D1R function (PD30) [194].
PD2–12 (Alc vapor, 4 h/day) (SD Rats)	23 mg/dL, pups	BLA	Decreased DA; Reduced D1R-mediated potentiation of sIPSCs; Impaired D3R-mediated suppression of mIPSCs (PD40–50) [197].
PD7 (2.5g/kg/twice) (C57 mice)	490 mg/dL, PD7	HP&NC	Enhanced AEA levels, NAPE-PLD, GDE & CB_1_ expression (PD7) [45].
GD8–20 (3 g/kg/twice a day) (SD rats)	281–341 mg/dL, maternal	VTA-DA	Impaired eCB-LTD (PD28–70) [198].
GD0–20 (Alc vapor) (C57 mice)	84 mg/dL, maternal	DLS-MSNs	Increased excitability of MS neurons; Increased eCB tone (PD90) [199].
GD8–20 & PD0–10 (3 g/kg/twice a day) (SD rats)	281–341 mg/dL, maternal	VTA-DA	Reduced CB_1_ mRNA expression (PD60–70) [200].
GD1–20 (Alc liquid diet) (SD Rats)	ND	MC & SSC	Reduced 5-HT, 5-HIAA &5-HT_1_ receptors (PD19) [184,201].
GD8–15 (Alc liquid diet) (C57 mice)	ND	MFB, MR, DR, HP, etc.	Reduced 5-HT (E15/18) [202,203,204].
GD15–20 (0.5–2g/kg/day) (SD Rats)	3.32–106 mg/dL, maternal	DR	Reduced 5-HT and TPH (PD37) [205].
GD1–20-PD20 (6% Alc) (CD1 mice)	73–102 mg/dL (PD21)	Amy&CC	Reduced 5-HT (Amy&CC); Reduced 5-HT1R (CC); Increased 5-HTR (Amy) (PD77–84) [206].
GD8–20 & PD0–10 (3 g/kg/twice a day) (SD rats)	281–341 mg/dL, maternal	VTA-DA	Enhanced the electrical activity of DRn 5-HT neurons (PD60–70) [200].

ND, not determined.

## Data Availability

Not applicable.

## Abbreviations

Alc, alcohol; FB, forebrain; SD, Sprague–Dawley; LE, Long-Evans; CE, cortex; WB, Whole brain; i.p, intraperitonial injection; GD, gestational day; PD, postnatal day; NC, neocortex; VTA, ventral tegmental area; mEPSCs, miniature excitatory postsynaptic currents; EPSCs, excitatory postsynaptic currents; mPFC, medial prefrontal cortex; WH, Wistar Han outbred rats; dHP, dorsal HP; A, albino rats; THA, thalmus; CBL, cerebellum, FC, frontal cortex; OLB, olfactory bulbs; Amy, Amygdala; AC, anterior colliculus; GAD, glutamate decarboxylase; mFC, medial frontal cortex; PV, parvalbumin; IN, interneurons; MS, medial septum; ACC, anterior cingulate cortex; SC, somatosensory cortex; MC, motor cortex; PN, pyramidal neurons; OFC, orbitofrontal cortex; IPSCs, inhibitory post-synaptic currents; PYN, pyramidal neurons; CR, calretinin; CB, calbindin; ST, striatum; PC, pyriform cortex; NC, neocortex; RSC, retrosplenial cortex; PPF, paired-pulse potentiation; LTP, long-term potentiation; LTD, long-term depression; HP, Hippocampus; NSDDA, nigrostriatal dopaminergic neurons; DA, dopamine; DL-ST, dorsolateral striatum; D1R, dopamine receptor 1; BLA, basolateral amygdala; D3R, dopamine receptor 3; NAPLE-PLD, N-Arachidonoyl phosphatidylethanolamine–phospholipase D; GDE1, glycerophosphodiesterase (GDE1); CB_1_, cannabinoid receptor type 1; eCB, endocannabinoids, DRn5-HT, dorsal raphe nucleus-serotonin neurons; DLS, dorsolateral striatum; MSNs, medium spiny neurons; MC, motor cortex; 5-HT, serotonin; 5-HIAA, 5-hydroxy indole acetic acid; SSC, somatosensory cortex; MFB, medial forebrain; MR, median raphe; DR, dorsal raphe; TPH, tryptophan hydroxylase; Amy, amygdala; CC, Cingular cortex, [^3^H]tritiated thymidine, MK801, Inhibitor of the N-Methyl-D-aspartate; CNS, central nervous system; FASD, Fetal alcohol spectrum disorder; MD, Medial Forebrain; FAS, fetal alcohol syndrome; AMPAR, α-amino-3-hydroxy-5-methyl-4-isoxazolepropionic acid receptor; *GABA*, gamma-aminobutyric acid; NMDAR, N-methyl-D-aspartate receptor; mGluRs, metabotropic glutamate receptors; iGluRs, ionotropic glutamate receptors; GluD, glutamate delta; PD, postnatal day; fEPSPs, field excitatory postsynaptic potentials; AEA, N-arachidonoylethanolamide or anandamide; 2-AG 2-arachidonylglycerol; eCBs, endocannabinoids; CB_1_; cannabinoid receptor type 1; VGCCs, N-type voltage-gated Ca^2+^ channels, FMI-43; KCl, potassium chloride; FMI-43, [N-(-3-trethylammoniumpropyl)-4-(-(dibutylamino) styryl) pyridinium dibromide; SR141716A, *N*-(Piperidin-1-yl)-5-(4-chlorophenyl)-1-(2,4-dichlorophenyl)-4-methyl-1*H*-pyrazole-3-carboxamide hydrochloride; GD, gestational day; GluR, glutamate receptor subunit; DG, dentate gyrus; Glu N, NMDA receptor subunit; mRNA, messenger ribonucleic acid; PSD95, Postsynaptic density protein 95; CREB, cAMP response element-binding protein; ERK, extracellular signal-regulated kinase; eEPSCs, evoked field excitatory postsynaptic potentials, LFS, low-frequency stimulation; AC, anterior colliculus; E, embryonic day; IPSC, inhibitory postsynaptic current; *GSX2* (GS Homeobox 2); NPY, Neuropeptide Y; SST, Somatostatin; DLX, Distal-Less Homeobox, WS, Wistar; A, Albino; AUD, alcohol use disorder.
